# Psychosocial factors associated with sickness absence in employees at a federal public university

**DOI:** 10.47626/1679-4435-2023-1191

**Published:** 2024-08-05

**Authors:** Mariana Valls Atz, Eduardo Remor

**Affiliations:** 1 Programa de Pós-Graduação em Psicologia, Instituto de Psicologia, Serviço Social, Saúde e Comunicação Humana, Universidade Federal do Rio Grande do Sul (UFRGS), Porto Alegre, RS, Brazil

**Keywords:** occupational health, absenteeism, occupational stress, universities, mental health, saúde do trabalhador, absenteísmo, estresse ocupacional, universidades, saúde mental

## Abstract

**Introduction:**

There is a need to understand which factors are associated with sickness absence in the context of public service in order to guide efforts to prevent illness in workers.

**Objectives:**

We investigated whether lifestyle, locus of health control, work-related stress, and self-perception of physical and mental health are associated with sickness absence from a biopsychosocial perspective.

**Methods:**

This is a cross-sectional study using an online questionnaire and the participants comprised 898 employees at a federal university. The assessment included instruments on sociodemographic and occupational characteristics, sickness absence, lifestyle (FANTASTIC Lifestyle scale), locus of control (Multidimensional Health Locus of Control Scale), workrelated stress (Health Safety Executive), and physical and mental health (Short-Form Health Survey 12 - version 2). A Poisson regression model was constructed using Generalized Estimating Equations to identify the variables associated with sickness absence, with a p ≤ 0.05 significance level.

**Results:**

We found that work-related stress, locus of control, physical and mental health, administrative or technical job role, female gender, and longer service time at the institution were associated with a higher number of days absent from work due to illness (for all associations, p < 0.001).

**Conclusions:**

The present study contributes to the literature by offering additional data on sickness absence in the context of Brazilian public university employees.

## Abstract (pt)

**Introdução:**

É necessário compreender quais fatores estão associados ao absenteísmo-doença no contexto do serviço público para direcionar a prevenção do adoecimento em trabalhadores.

**Objetivos:**

Foi investigado se estilo de vida, lócus de controle da saúde, estresse relacionado ao trabalho e autopercepção de saúde física e mental estão associados com absenteísmodoença, a partir de uma perspectiva biopsicossocial.

**Métodos:**

Trata-se de um estudo transversal por meio de questionário *online*. Participaram 898 servidores públicos de uma universidade federal. Utilizaram-se cinco instrumentos: um questionário de características sociodemográficas e ocupacionais; a escala FANTASTIC Lifestyle, para avaliação do estilo de vida; a escala Multidimensional Health Locus of Control Scale, que avalia lócus de controle da saúde; as diretrizes do Health and Safety Executive, para avaliação do estresse relacionado ao trabalho; e o Short-Form Health Survey 12 - *version* 2, para avaliação da saúde física e mental. Para identificar as variáveis associadas ao absenteísmo-doença, foi empregado o modelo de regressão de Poisson através de equações de estimativas generalizadas, sendo considerado o nível de significância p ≤ 0,05.

**Resultados:**

Apontou-se que maior estresse relacionado ao trabalho, lócus de controle da saúde, pior autopercepção de saúde física e mental, técnicos administrativos, gênero feminino e maior tempo de serviço na instituição se associam com mais dias de ausência ao trabalho por motivo de doença (para todas as associações: p < 0,001).

**Conclusões:**

O presente estudo contribui com a literatura ao oferecer dados adicionais sobre absenteísmo-doença no contexto de serviço público universitário brasileiro.

## INTRODUCTION

Sickness absence is an important indicator in occupational health, constituting absence from work caused by the worker’s incapacity to work, excluding pregnancy and incarceration. It may be caused by an accidental disease or injury, may be a measure to avoid the spread of transmissible diseases, or could be because of conditions varying from a mild ailment to a serious disease.^1^ Sickness absence cannot be explained by health problems only, since its determinants are multiple and complex. Along the same lines, the interface between health and work is not limited to aspects of working conditions, but includes a wide range of factors. Some of these are parallel to work, operate on the occupational health and disease process, and, when identified, can be important to promotion of workers’ health.

Lifestyle is increasingly identified as a risk factor for a wide range of conditions of physical health. Many elements of an unhealthy lifestyle appear to be associated with sickness absence, although there is no consensus in the literature on which of them are associated. For example: smoking, alcohol use, frequency of physical activity, and obesity have been associated with sickness absence in some studies,^2-4^ but other studies did not find significant or consistent results for the same variables.^5-7^ With the objective of contributing to the literature, it is of interest to determine whether lifestyle as a general measure, rather than specific aspects of it, might be associated with sickness absence. This study therefore includes individuals’ perceptions of their own lifestyles.

In the occupational setting, promotion of healthy environments encompasses a series of policies and activities in the workplace, conceived to allow employers and workers of all levels to increase their control over their health and enable them to improve it. Thus, one measure that could be used to assess beliefs about control is the health locus of control, a construct understood as people’s perception of who or what has control over their health. It is manifest as a tendency to perceive life events as controlled by oneself, in which case the locus of control is internal, or as controlled by other factors, beyond oneself, such as luck or other people - doctors and priests, for example.

In the context of occupational health, this construct can be related in a number of ways. For example, it appears that workers with a higher internal health locus of control more often use the results of regular checkups to manage their own health.^8^ Moreover, among healthy workers, an external locus of control was associated with absences from work of up to 2 weeks, and in workers with a diagnosis of depression and/or anxiety it was associated with absences from work for more than 2 weeks.^9^ For this reason, this variable was included in the present study, in order to determine whether beliefs about control over health are associated with sickness absence among university employees.

However, there are health determinants that are more or less under the worker’s control, such as the psychosocial risks of their work. These are linked to the characteristics of their tasks, the organization, their employment, and their time in service, and analysis shows that they can have negative effects on workers’ health, as is the case with occupational stress, which has been associated with sickness absence. For example, it appears that there is a significant relationship between the number of days off work for sickness and different dimensions of occupational stress, such as workload, conflicting roles, the physical environment, and total stress.^10^

In public university settings, intensification of the working day, overload, and overlapping activities are stress factors associated with mental illness among faculty members.^11^ Among public university technical and administrative staff, inadequate work infrastructure and low social support have been identified as important aspects in occupational stress.^12^ Therefore, this study also investigated the contribution of occupational stress to the phenomenon of sickness absence in the university setting.

Other factors may also be related to the phenomenon studied, including self-perception of occupational health, since it is expected that, if there is sickness, there will be worse health self-assessment. It is important to understand the relationships between psychosocial aspects of working in a public university and sickness absence, so that the occupational health field can establish health indicators and actions for promotion and prevention that are aligned with the setting. This could contribute to definition of priorities for where to intervene in terms of occupational health promotion. Therefore, the objective of this study was to investigate whether lifestyle, health locus of control, work-related stress, and self-perception of physical and mental health are associated with sickness absence in a public university setting.

## METHODS

### PARTICIPANTS

The target-population of the study was public sector employees at a Federal university in the South of Brazil, which, at the time, had around 5,470 employees, counting faculty and technical and administrative staff. With regard to the general characteristics of this population: 53.8% were faculty members; 52% had a doctorate; 34.2% had been at the institution for 3 to 9 years; and 52% were men.

The study inclusion criteria were consenting to take part in the study; being an active employee working at the institution studied; and completing the questionnaire. Exclusion criteria were defined as follows: being on study leave; being on technical secondment or loan to another institution for more than 90 days; and having taken maternity leave during the previous 12 months. From the overall population, considering the inclusion and exclusion criteria, a total of 898 employees were recruited to the study sample anonymously and voluntarily. Of these participants, 59.5% were women, 53.2% were technical and administrative staff, and 49.6% had a doctorate, of whom 90.4% were faculty members. This sample has 99% confidence and a 4% margin of error.

### STUDY DESIGN AND PROCEDURES

This is a cross-sectional study employing a descriptive quantitative method based on a survey with questionnaires. Data collection was performed using the online platform Survey Monkey^®^ (https://pt.surveymonkey.com/). All of the university’s employees were invited to take part via the institution’s human resources department’s e-mail, after prior authorization from the academic authorities. In addition to the initial invitation, a further 3 reminder e-mails were sent over the 3-month data collection period, which began on January 28 and ended on April 17, 2020. It should be noted that the sample is non-probabilistic, since selection was based on employees’ availability and inclination to complete the questionnaire.

### VARIABLES AND INSTRUMENTS

#### Sociodemographic and occupational characteristics

These data were collected using a questionnaire developed *ad hoc* for this study. The items assessed were age, gender, race, job type (faculty member/technical and administrative staff), educational level, time in service at the institution, and management job role.

#### Lifestyle

Assessed using the FANTASTIC Lifestyle scale, which covers several layers of health habits. Developed in the 1980s,^13^ this is a 5-item instrument and the lower the subject’s score on a scale from 0 to 100, the greater the need for changes in lifestyle habits. The instrument has been adapted and validated for Brazil.^14^

#### Health locus of control

Assessed using the Multidimensional Health Locus of Control Scale (MHLC), version A.^15^ This instrument comprises 18 items, with three subscales: internality, powerful others, and chance. The three types of health locus of control were treated independently in this study. For each type, the scores vary from 0 to 30, where a high score on each subscale is indicative of the belief that health is controlled by that factor (individual control, by other people, or by luck). This instrument had been validated for Brazil.^16^

#### Work-related stress

Assessed using the Health and Safety Executive - Indicator Tool (HSE-IT). This instrument comprises 35 items, distributed in seven dimensions related to psychosocial factors of work. Responses are given on a Likert type scale and it is used as a measure of workrelated stress.^17^ The higher the score, the lower the stress that the respondent is exposed to because of psychosocial factors of work. This instrument has been translated and validated for Brazil,^18^ demonstrating adequate psychometric properties for use in research.

#### Self-perceived health

Assessed using the Short-Form Health Survey 12 - version 2 (SF-12v2), which is a self-report measure of health-related quality of life.^19^ Two summary subscales can be derived from the instrument, one for mental health and one for physical health. Scores for each scale range from 0 to 100, where the higher the score, the better the self-perception of health. This instrument has been translated and validated for Brazil, demonstrating adequate psychometric properties.^20^ Permission to administer the instrument was obtained.

#### Sickness absence

Sickness absence was measured using the following single open question “During the last 12 months, how many days were you absent from work for reasons of health?” It should be noted that the question does not only cover absence for ill health, but also any absences for health reasons, such as for medical tests and consultations.

### ETHICAL CONSIDERATIONS

This study complies with the guidelines set out in Resolution 466, of December 12, 2012, regulating the ethics of research with human beings. This study was approved by the Research Ethics Committee at the Instituto de Psicologia da Universidade Federal do Rio

Grande do Sul (Ethics Appraisal Submission Certificate 27808619.6.0000.5334). Participation was anonymous and data were only used for statistical analyses. The free and informed consent form provided information on the study objectives, data collection methods, risks and benefits, and participants’ right to drop out at any time.

### DATA ANALYSIS

Statistical analysis was conducted using the Statistical Package for the Social Sciences, version 25.0 (SPSS Inc., Chicago, IL, USA, 2018). Results are presented using descriptive statistics for absolute and relative distributions (n - %) and measures of central tendency (mean and median) and variability (standard deviation and interquartile ranges). Symmetry of continuous distributions was assessed using the KolmogorovSmirnov test. A statistical significance level of 0.05 was adopted for all analyses.

Variables were compared in relation to sociodemographic and occupational characteristics. Where comparisons involved two groups, the MannWhitney U test was used, because the variable sickness absence had an asymmetrical distribution. For this analysis, the effect size r was calculated, ranging from 0 to around 1. For this calculation, 0.10 to 0.29 is considered a small effect size; 0.30 to 0.50 is considered moderate; and greater than 0.5 is considered large. When comparisons of scores involved three or more groups, the KruskalWallis test and Dunn’s post-hoc test were used and effect sizes were calculated using epsilon-squared (ɛ^2^), where values from 0.01 to 0.08 are considered a small effect size; 0.08 to 0.26 are moderate; and greater than or equal to 0.26 is large.^21^

A Poisson regression model with robust variance was used to identify variables with the greatest association with sickness absence, constructed using generalized estimating equations. The model included all independent variables that exhibited a minimum level of significance less than or equal to 0.05 in the bivariate analysis.

### NOTE ON THE IMPACT OF COVID-19

Part of the research project was conducted during the initial phase of the COVID-19 outbreak, which was declared a pandemic by the World Health Organization (WHO) in March of 2020. Data were compared between two subsets to check for any impact on responses caused by the pandemic: one comprising data collected before measures to prevent contagion were initiated at the university, i.e. from January 28 to March 17 (n = 726), and the other comprising data from March 24 to April 17 (n = 172). Since the two subsets were of unequal size, differences in variables between the two groups were analyzed using the Mann-Whitney nonparametric test for independent samples. It was decided to maintain the second subset in the sample since the effect sizes in all analyses were smaller than r = 0.1.

## RESULTS

Table 1 lists the descriptive data for the sample in absolute and relative terms. In relation to gender, women predominated (59.8%). The most prevalent job type was administrative and technical (53%). The most prevalent time in service categories were 3 to 9 years (31.3%), and 10 to 19 years (21.5%).

**Table 1:** Absolute and relative distributions of sociodemographic and occupational characteristics for the sample

Variables	Sample (n = 898)^*^	
	n	%
Gender		
Female	543	60.5
Male	355	39.5
Age categories (years)		
19-24	7	0.8
25-29	55	6.1
30-39	274	30.5
40-49	214	23.8
50-59	241	26.8
60-64	74	8.2
65 or over	33	3.7
Race/skin color		
Black	36	4.0
Brown	47	5.2
White	803	89.4
Yellow	9	1.0
Indigenous	3	0.3
Educational level		
Secondary education	20	2.2
**n**	%
Technical college	20	2.2
Higher education	100	11.1
Postgraduate certificate	161	17.9
Master’s degree	150	16.7
Doctorate	447	49.8
Job type		
Faculty member	422	47.0
Administrative and technical staff	476	53.0
Time in service at the institution (years)		
Up to 3	185	20.6
3-9	281	31.3
10-19	193	21.5
20-29	132	14.7
30-34	51	5.7
35 or more	56	6.2
Currently in a management role?		
Manager	213	23.7
Subordinate	685	76.3

* Percentages based on entire sample.

The information on sickness absence showed that the number of days absent ranged from 0 to 240. The median was 1 day and 75% of the sample reported 5 days absent or fewer. In relative terms, 43.2% reported no absence from work, and 31.7% reported from 1 to 5 days absent, as shown in Table 2.

**Table 2:** Measures of central tendency and variability for days of sickness absence and absolute and relative distribution for sickness absence

Sickness absence	Sample (n = 898)	
	n	%
Absenteeism (number of days reported)		
Mean ± standard deviation	6.7 ± 176 (0-240)	
Median (interquartile range)	1.0 (00-5.3)	
Absenteeism (classification)^*^ (days)		
None	388	43.2
1-5	285	31.7
6-15	123	13.7
16-30	64	7.1
31-90	30	3.3
More than 90	8	0.9

* Percentages based on entire sample.

Sickness absence was analyzed in terms of sociodemographic and occupational variables, as shown in Table 3. There was a statistically significant difference between genders (p < 0.001), revealing that the median number of days of absence was greater among women (median [interquartile range)]: 2.0 [0.0-8.0]). Technical and administrative staff (median [interquartile range]: 3.0 [0.0-10.0]) had a significantly higher number of days absent (p < 0.001) than faculty members.

**Table 3:** Days of sickness absence by sociodemographic and occupational profile characteristics in public university employees

Variables	Sickness absence							
	n	Mean	SD	Quartiles			Test statistic (P)	Effect size
				Q1	Q2 (median)	Q3		
Gender Female	543	7.6	15.9	0.0	2.0	8.0	Z_MN_ = -6446 MW (p = 0.001^†^)	0.215^*^
Male	355	5.3	19.8	0.0	0.0	3.0		
Age (categories) (years) 19-24	7	3.6	4.3	0.0	1.0	9.0	H_KW_= 11.736 (p = 0.039^§^)	0.079^*^
25-29	55	4.1	6.9	0.0	2.0	5.0		
30-39	274	6.2	12.1	0.0	2.0	7.0		
40-49	214	6.7	16.8	0.0	1.0	6.0		
50-59	241	7.9	23.1	0.0	2.0	5.0		
60-64	74	6.3	19.8	0.0	0.0	3.0		
65 or over	33	7.5	22.7	0.0	2.0	2.5		
Race/color Black	36	14.3	33.6	0.0	1.0	13.8	H_KW_= 1.152 (p = 0.886^§^)	
Brown	47	5.1	10.1	0.0	0.0	6.0		0.065^*^
White	803	6.5	17.0	0.0	1.0	5.0		
Yellow	9	2.9	3.3	0.0	2.0	5.0		
Indigenous	3	4.0	5.3	0.0	2.0	10.0		
Educational level							H_KW_ =	
Secondary education Technical college	20 20	3.5 3.5	4.5 7.0	0.0 0.0	1.0 0.5	5.5 4.5	10.055 (p = 0.040^§^)	
Higher education	100	6.3	11.1	0.0	2.0	8.8		
Postgraduate certificate	161	10.6	23.7	0.0	3.0	10.0		0.092^*^
Master’s degree	150	9.8	18.9	0.8	3.0	12.0		
Doctorate	447	4.6	16.0	0.0	0.0	3.0		
Job type Faculty member Administrative and technical staff	422 476	4.0 9.0	15.9 18.6	0.0 0.0	0.0 3.0	2.0 10.0	Z_MW_ -10.603 (< 0.001^§^)	0.353^*^
Time in service at the institution (years) Up to 3	185	3.4	8.3	0.0	0.0	3.0	H_KW_= 32.295 (< 0.001^§^)	
3-9	281	6.0	13.2	0.0	2.0	7.0		
10-19	193	9.6	20.0	0.0	2.0	10.0		0.058^*^
20-29	132	10.6	30.4	0.0	0.0	5.8		
30-34	51	4.7	13.6	0.0	0.0	4.0		
35 or more	56	3.5	*12*	0.0	0.0	3.0		
Currently in a management role?							Z_MW_ = 2.583 MW (p = 0.108^*^)	0.086^*^
Manager	213	5.0	14.6	0.0	0.0	4.5		
Subordinate	685	*12*	18.4	0.0	1.0	6.0		

r calculated for the Mann-Whitney U test; ^†^ Mann-Whitney U test;

‡ ɛ^2^ calculated for the Kruskal-Wallis test; § Kruskal-Wallis test Dunn’s post-hoc test. SD = standard deviation.

The initial exploratory hypothesis was that all of the variables analyzed would be associated with sickness absence. The Poisson regression model with robust variance constructed with generalized estimating equations was used to identify the variables with the strongest associations with absenteeism.

Initially, the variables lifestyle, health locus of control, work-related stress, physical health, mental health, and those categorical variables that had been significant in the previous bivariate analysis were included in the model. Lifestyle had a weak relationship with sickness absence, with behavior that oscillated (depending on the variables tested, the model identified it as a protection factor or risk factor, in all cases with an almost insignificant prevalence ratio [PR]) and lost explanatory power with relation to the other explanatory variables. Specific items from the lifestyle instrument were also tested, as indicated by analysis of the literature, such as obesity, nutrition, and physical activity, but none of them proved to be significant. Therefore, lifestyle was removed from the model.

As shown in Table 4, the strongest association was observed with time in service at the institution, where the probability of employees with 20 to 29 years at the institution having more days absent was 3.610 (95%CI PR: 3.103-4.200) times that of individuals who had 35 years or more in service.

**Table 4:** Poisson regression model for sickness absence among public university employees

Variables	Regression coefficient			PR		
	B	Standard error	P value p	PR	95%CI (PR)	
(Intercept)	3.887	0.1719	0.000	48.771	34.819	68.312
Gender Female	0.169	0.0298	0.000	1.184	1.117	1.255
Male (Ref)				1	-	-
Time in service at the institution (years) Up to 3	-0.050	0.0828	0.545	0.951	0.809	1.119
3-9	0.435	0.0768	0.000	1.544	1.329	1.796
10-19	0.890	0.0764	0.000	2.436	2.097	2.829
20-29	1.284	0.0772	0.000	3.610	3.103	4.200
30-34	0.446	0.0967	0.000	1.562	1.292	1.888
35 or more (Ref) Job type				1	-	-
Faculty member				1	-	-
Administrative and technical staff (Ref) Health locus of control	0.903	0.0688	0.000	2.156	2.054	2.377
Internality	0.029	0.0040	0.000	1.029	1.021	1.038
Powerful others	0.022	0.0034	0.000	1.022	1.015	1.029
Chance Self-perceived health	0.049	0.0038	0.000	1.051	1.043	1.058
Physical	-0.050	0.0017	0.000	0.952	0.949	0.955
Mental	-0.021	0.0014	0.000	0.980	0.977	0.982
Work-related stress	-0.204	0.0272	0.000	0.815	0.773	0.860

Dependent variable: Sickness absence. Model: intercept, gender, time in service, job type, internality, powerful others, chance, physical health (higher score, better health), mental health, work-related stress (higher score, lower stress).

95%CI = 95% confidence interval; PR = prevalence ratio; Ref = reference variable.

Job type was also associated with sickness absence, showing that the probability of technical and administrative staff taking more days absent from work because of sickness was 2.156 (95%CI PR: 2.054-2.377) greater than for faculty members. Higher numbers of days absent were also related to female gender, which had a 1.184 (95%CI PR: 1.117-1.255) times greater probability of absence from work because of illness, when compared to male gender. Although it had been significant in the bivariate analysis, the variable gender lost strength of association in the model, with a less significant result.

The results also showed that higher scores on the three subscales of the MHLC were associated with higher probabilities of sickness absence: internality (PR: 1.029; 95%CI PR: 1.021-1.038), powerful others (PR: 1.022; 95%CI PR: 1.015-1.029), and chance (PR: 1.051; 95%CI PR: 1.043-10.58). The most representative dimension was chance, where an increase of one unit in the score for this dimension was associated with a 5.1% greater probability of more days absent.

The model defined the health subscales as protection factors, showing that low scores for physical health (PR: 0.952; 95%CI PR: 0.949-0.955) and mental health (PR: 0.980; 95%CI PR: 0.977-0.982), i.e., worse physical and mental health assessments, were associated with more sickness absence days.

The non-categorical variable with the strongest association was work-related stress (PR: 0.815; 95%CI PR: 0.773-0.860), where lower scores on the instrument (i.e., greater stress) were associated with greater probability of absence from work for reasons of sickness. In other words, presence of occupational stress is a risk factor for sickness absence.

## DISCUSSION

Sickness absence is investigated within many different disciplines and in the context of many different research problems and there is a need to understand the contribution that different factors make to this phenomenon. This study contributes to this goal, since it observed that there was a higher probability of reporting sickness absence among mid-career employees, women, technical and administrative staff, and those with worse perceived physical health and mental health and work-related stress. A greater probability of sickness absence was also observed among those with an elevated score for one of the health loci of control, primarily external chance.

With regard to time in service at the institution, the up to 3 years category was not significant, suggesting that the probation period is not a risk factor for sickness absence. Longer periods of service were linked with a growing probability of sickness absence. This corroborates other studies that have shown that absenteeism rates are low during probation, but tend to increase once stability in public employment has been achieved.^22^

Compared with employees with 35 years or more in service, it was observed that there was an increasing risk of sickness absence up to 30 years into the career, when the probability of employee absence began to fall. This could suggest that from 3 years in service at the institution onwards, the probability of sickness absence begins to increase, which could be, for example, because of worsening of chronic diseases over time, or because employees are worn down by the job itself. Moreover, employees who continue working in the public sector after attaining the right to retire tend to have lower risk of sickness absence, suggesting that only those in good health continue working.

In regard to job type, the results showed that technical and administrative staff had a 2.15 times greater probability of sickness absence than faculty, which corroborates other studies in similar settings.^23,24^ It is relevant to point out that there are different systems for controlling attendance at the institution analyzed: technical and administrative staff clock in with an electronic time clock, whereas faculty members do not. The results could therefore be biased by a tendency for faculty members to under report (because of forgetfulness and less control over absence, for example), rather than there necessarily being a lower rate of occupational sickness. This result may also reflect that faculty members have greater flexibility to plan their activities, making their own perceptions of absence from work for short periods more confused. Future studies should investigate this issue to better elucidate it.

The results show that women tend to be absent for health reasons more often than men. This corroborates other studies in similar settings^23-25^ and the literature indicates some reasons for this, such as gender stereotypes and women working double shifts because of unseen domestic work.^26^ There may also be a tendency for women to attend health checkups more frequently than men. The importance of gender equality and women’s health policies within federal public employment should therefore be emphasized.

Special attention should be paid to the results for health locus of control. The literature has suggested that there is a relationship between beliefs about control and time off from work for health reasons, but there is evidence that the impacts of these beliefs on the outcome sickness absence are not uniform across different workers.^27^ The present study contributes to the discussion in the literature by demonstrating that all three subtypes of health locus of control - internal, chance, and powerful others - are associated with more days of absence from work for health reasons. This could indicate that any of these conditions, in an extreme form, constitutes a risk factor for greater time absent, emphasizing the need for a balance between the three types of health locus of control. On the other hand, the result could also indicate that this variable has a heterogeneous impact on employees, depending on other characteristics of the setting and of personality that were not analyzed in the study.

With regard to self-perception of physical and mental health, it was observed that the lower the scores on the instrument, the greater the probability of the employee reporting sickness absence. This corroborates the literature, since other studies have already identified an association between self-assessed health and sickness absence.^28^ This result supports this association in a Brazilian public university setting and, in institutional terms, contributes by presenting a self-report instrument that could be useful for identifying workers at risk of prolonged sickness absence.

Lifestyle was removed from the model because its behavior in the analyses was oscillating, with weak associations. In general, the literature has explored the relationship between certain lifestyle habits and sickness absence, but there is still a lack of consensus on this subject. It appears that lifestyle factors are associated with absence for different types of health reasons depending on the pathology, but it is still difficult to determine the nature of these associations.^29^ In contrast with other investigations, in the present study, the decision was taken to analyze the general perception of lifestyle. The results suggest that perceived lifestyle was unrelated to the number of days of absence from work for health reasons in this population. This result could have been influenced by the type of measure employed, but could also indicate that perceived lifestyle has heterogeneous impacts on different segments of the population, depending on other factors that were not assessed in this study.

Finally, the results suggest that there was a higher probability of more days absent from work when perceived work-related stress is worse, and this variable, together with time in service, had the strongest association. Previous studies have already highlighted occupational stress as a risk factor among both university faculty members and technical and administrative staff.^11,12^

One of this study’s strong points was to show that stress related to the psychosocial risks of work had greater weight than individual characteristics such as lifestyle and beliefs about health control. In view of this, it is suggested that in order to reduce sickness absence, actions that attenuate occupational stress factors (greater organizational support and better working conditions, for example) would be more effective than actions merely focused on changing health habits. The importance of taking exposure to work-related stress into account as a risk factor for sickness absence is therefore emphasized, as is the importance of action aimed to mitigate occupational stress and improve the work environment in public university settings.

Certain limitations of this investigation should be mentioned. Since it is a cross-sectional study, it cannot help to understand the long-term effects on sickness absence of the variables analyzed. Moreover, characteristics of the study could be sources of bias, such as the exclusive use of self-report measures, which may not be sufficiently adequate, and also the sample type.

Additionally, the limitations of exclusive use of quantitative instruments to assess occupational health are well known as is the need to take a wider and integral perspective on occupational health and the factors involved in sickness in order to improve this.^30^ Notwithstanding these limitations, this study contributes to the literature by identifying factors associated with sickness absence in a Brazilian public university setting.

It would be remiss to fail to reflect on the COVID-19 pandemic and remote working. The concept of sickness absence was defined on the basis of a model of working in the workplace and there are issues related to how to approach it in the current scenario. It is believed that rather than the pandemic reducing the adequacy of the results, the study actually throws light on a problem that is at risk of becoming invisible and hidden by the disruptive transformations inherent to the transition from a workplace model of work to one that is virtual and occurs at home.

Detection of sickness absence, whether by the organization or by the worker, is more complex within the new models of work, but it remains a key problem in understanding occupational sickness. As such, in addition to identifying factors associated with the phenomenon, this study emphasizes the importance of considering the problem within the scope of occupational health.
